# A Scoping Review of Tobacco Control Health Communication in Africa: Moving towards Involving Young People

**DOI:** 10.3390/ijerph21030259

**Published:** 2024-02-23

**Authors:** Charity Aienobe-Asekharen, Emma Norris, Wendy Martin

**Affiliations:** Department of Health Sciences, Brunel University London, London UB8 3PH, UK; charity.aienobe-asekharen2@brunel.ac.uk (C.A.-A.); emma.norris@brunel.ac.uk (E.N.)

**Keywords:** tobacco control, smoking, health communication, campaigns, WHO FCTC, young people, Africa

## Abstract

Health communication has been highlighted as a cost-effective preventive intervention in Africa, where the prevalence of tobacco use is still relatively low compared to other World Health Organization (WHO) regions. This scoping review aimed to examine tobacco control health communication interventions in Africa. The review was guided by the PRISMA-ScR checklist. Data was extracted from 20 peer-reviewed papers, WHO Global Health Observatory on anti-tobacco mass-media campaigns for 54 African countries, and 6 WHO Framework Convention on Tobacco Control reports on Article 12. Data extraction informed by the Joanna Briggs Institute (JBI) data-extraction questions was used for peer-reviewed studies while a pre-determined template was used for the other sources. Narrative data synthesis informed by the JBI manual for evidence synthesis was employed. A lack of research that comprehensively addresses all areas of health communication and inconsistent use of health communication campaigns were identified. Only an average of 6 countries had ever implemented high-quality national mass-media campaigns in a decade, while an average of 33 countries consistently failed to conduct campaigns that lasted more than 3 weeks. Although the involvement of key populations was clearly vital to ensure content relevance and message clarity, a lack of health communication informed by young people was observed, as they rarely participated in key decision-making despite reportedly being the targets of interventions. Clear health communication for tobacco-use prevention informed by young people is lacking in African countries. Active participation of young people in developing targeted campaigns is needed to facilitate content relevance and comprehension to ultimately contribute to tobacco-use prevention.

## 1. Introduction

The deaths of over eight million people annually are attributed to tobacco use [1]. The burden of tobacco-related deaths and ailments is highest among people living in low- and middle-income countries where 80% of the world’s 1.3 billion tobacco users reside [1]. Africa has been predicted as the continent that will have the highest increase in smoking prevalence without preventive measures being engaged [2,3,4]. It is estimated that in the absence of tobacco control measures, there will be an increase in tobacco consumption by almost 39% by 2030 [2,4]. This is estimated to be the highest expected regional increase globally [2].

According to the 2022 Atlas of African Health Statistics by the WHO Regional Office for Africa [5], the African region has a lower prevalence of smoking at 10%, in contrast to higher rates of 16% in the Americas and 29% in South-East Asia. Given Africa’s projected population boom [6,7], and a rapid increase in tobacco use [2], health communication as a cost-effective preventive approach is recommended as one of the best buys [8,9]. 

Health communication is both a science and an art that seeks to advance the health and well-being of populations through communication [10]. Due to the multidisciplinary nature of health communication, there are several definitions and overlaps [11] (pp. 8–9). The majority of definitions point to the role of health communication in influencing, supporting, and empowering individuals, communities, healthcare professionals, policymakers, or special groups, to adopt and sustain a behavior or a social, organizational, and policy change. This change will ultimately improve individual, community, and public health outcomes [11] (p. 9). To empower populations to make the best decisions to achieve the best outcomes, health communication is often practiced in active collaboration with the focal population [11] (p. 10). 

Health communication interventions have been shown to address social norms, prevent smoking initiation, encourage quit attempts, and support policy [8,12]. These interventions are aimed at improving knowledge, addressing perceptions, and changing behavior [12]. The interventions are also aimed at ensuring information is effectively communicated at a level that people can understand, while engaging them in the decision-making process [13]. This is vital as targeted communication informed by the focal group is valuable, enhances the impact of health information, and increases relevance [14,15]. Health communication campaigns have been identified as economical, effective in preventing youth initiation and reducing tobacco use [8,12,16], and vital in the de-normalization of the tobacco industry, and de-glamorization of tobacco use [17]. 

The Florida anti-tobacco truth campaign that specifically targeted young people was reported as largely successful [18,19]. The campaign comprised of consistent and focused advertisements (2–3 on TV), which gave young people a sense of empowerment and projected that young people themselves needed to take action to put a stop to the tactic employed by the tobacco companies. The message was also clearly visualized—tobacco use can cause death and the tobacco industry is manipulative and needs to be stopped. In Africa, the SKY Girls campaign in Ghana in 2017 also recorded success as they engaged young people in the implementation phase using mass-media and social-media channels to de-glamorize tobacco use and to promote a positive sense of identity not defined by smoking [20,21]. 

While tobacco control health communication through the media is predicted to be the future battlefield for supremacy on tobacco narratives [22], tobacco control has not enjoyed media coverage even though the media are uniquely placed to influence the public health agenda and public opinion [23]. 

In Africa, the utilization of health communication as a tool for preventing smoking is still in its formative years and is fraught with several challenges. This is because there are limited studies that address the six different areas of health communication as highlighted by Schiavo [11] (pp. 26–28), including limited research that specifically targets the tobacco control health communication needs of young people aged 10–24 years as defined by the WHO [24]. In addition, tobacco control policy implementation is not homogenous across regions and could be impaired due to poor funding, poor policy enforcement, lack of government commitment, limited capacity for enforcement, tobacco industry interference, or the presence of cross-border marketing activities [25,26].

The need for a robust approach towards tobacco control health communication in Africa is more relevant now than ever. This is because the prevalence of tobacco smoking, though still low, will increase [2,27] as the continent moves from being a tobacco production hub to a tobacco consumption centre [2,28].

The growing population in Africa is expected to significantly increase by 2050 as one in every three births will be in Africa [6]. This projected population increase will necessitate interventions that are specifically targeted at young people. These targeted interventions would facilitate a reduction in tobacco-related diseases and deaths, and reduce out-of-pocket health expenditures, including contributing to the achievement of the Sustainable Development Goal 3 [29], specifically, target 3.4 and 3.a.

This review aims to highlight what has been implemented around tobacco control health communication within Africa with a particular focus on the involvement of young people by taking a scoping review approach to better facilitate a balanced view. A scoping review is necessary because tobacco control health communication in Africa is still in its infancy, with limited peer-reviewed literature. As such, utilizing grey literature and the appropriate supporting policy (WHO FCTC Article 12) will enable a more holistic framing of the present reality to inform future interventions. 

The objective of this review is to:

A. Identity the types of tobacco control health communication interventions in Africa.
B. Identify how young people are being involved in tobacco control health communication.

## 2. Method 

### 2.1. Data Sources and Identification

This review was guided by the PRISMA-ScR (Preferred Reporting Items for Systematic Reviews and Meta-Analyses Extension for Scoping Reviews) checklist [30]; a protocol was not registered, and critical appraisal was not conducted on identified papers (See Appendix A, Table A1). This was because the review aimed to identify and map all available evidence on tobacco control health communication in Africa, and to examine how young people were involved. 

Five databases (PsycInfo, PubMed, African Journals Online, Web of Science, and WHO Global Health Observatory on anti-tobacco mass-media campaigns) and six WHO FCTC Global Progress Reports were identified as sources of information. The databases recurring consistently across systematic reviews on tobacco control in Africa were identified for the literature search (PsycInfo, PubMed, and African Journals Online) while an additional search was conducted in Web of Science. 

For the grey sources, the WHO Global Health Observatory on anti-tobacco mass-media campaigns (WHO GHO ATMCs) was identified and traced from key papers that referred to the WHO FCTC and MPOWER strategy [4,22,25,26]. The WHO FCTC Global Progress Report on Article 12 was also identified from papers that referred to the pivotal role of the WHO FCTC in facilitating tobacco control [22,25,26,27]. All data sources in this review were accessed between 4 January 2021 and 17 November 2023. 

**Figure 1:**
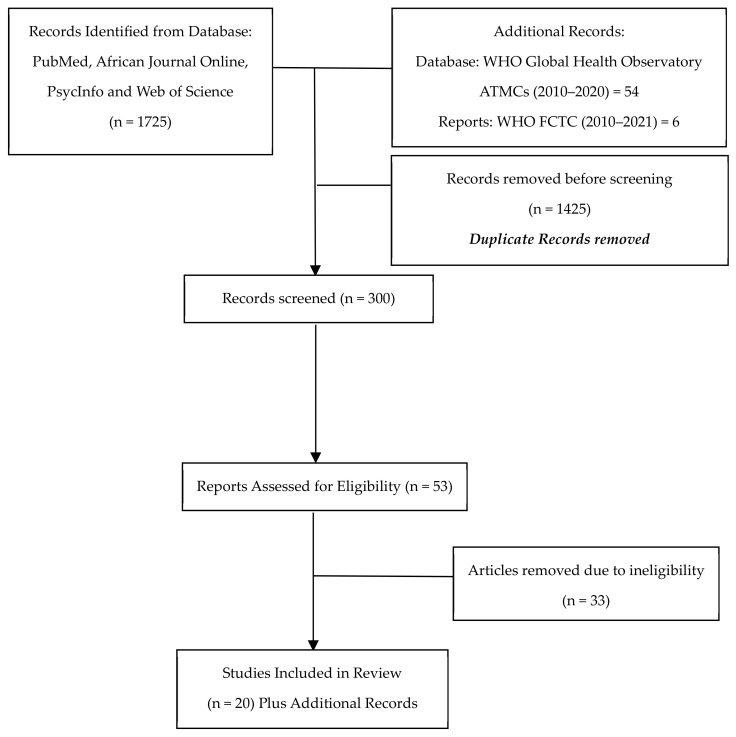
Sources of evidence.

No time restriction was applied during the database search. Search terms used include: Health AND (Message OR Campaign OR Communication) AND (Nicotine OR Tobacco) AND Africa, Health AND (Message OR Campaign OR Communication OR Awareness OR Training) AND (Nicotine OR Tobacco) AND Africa. The highlighted search string was further used specifically for each of the identified 54 African countries in the WHO GHO ATMCs to ensure key papers were not missed. A summary of the sources is highlighted in Figure 1.

### 2.2. Eligibility Criteria

The authors assessed studies as eligible if they focused on any of the six areas of health communication as acknowledged by Schiavo [11] (pp. 26–28), and/or focused on health communication as highlighted by the focus of the WHO FCTC Article 12 [31]. The classification offered by Schiavo covers varied aspects of health communication. The authors choose this classification as it offers a clear yet broad perspective on the different areas of health communication.

The six areas of health communication are:

A. Interpersonal communication.
B. Mass media and new media communication.
C. Community mobilization and citizen engagement.
D. Professional medical communications.
E. Constituency relations and strategic partnerships in health communication.
F. Policy communication and public advocacy.

Studies were included if they were conducted among individuals living in Africa, not Africans in the diaspora, and were targeted at young people 10–24 years old. The authors included studies that had the full-text available, were written in the English language, and did not combine tobacco and other substances like cannabis and alcohol. Studies that focused on forms of health communication not (yet) attached to a tobacco product were also a primary focus, as these would provide insight into the involvement of participants.

### 2.3. Search Approach and Characteristics of Sources

Guided by the six areas of health communication and the WHO FCTC in the eligibility criteria, studies were identified from the literature search and included in the review. Health communication interventions reported in the WHO Global Health Observatory and the WHO FCTC Article 12 Reports were also accessed online as highlighted below:

A. Database: The WHO GHO ATMCs data for 54 African countries from 2010–2020

The WHO Global Health Observatory (GHO) is an online database hosting data segmented into thirty-three themes [32]. Within the tobacco control theme, there are eight sub-themes, one of which is anti-tobacco mass-media campaigns. These campaigns are focused on warning people about the dangers of tobacco use, using varied channels of communication. In the WHO GHO, anti-tobacco mass-media campaigns are classified per country data into five categories based on the seven characteristics (CTS) of a high-quality campaign. These categories and characteristics have been highlighted below in Table 1. The number of characteristics identified in a county’s campaign is used to identify the category under which the campaign will be rated. More details on the data per country can be found in Appendix A or obtained directly from the WHO GHO website.

B. Reports: Six reports from the WHO FCTC Global Progress Report on Article 12 from 2010–2021

The WHO Global Progress Report is a biennial report from the WHO FCTC parties collated by the WHO [33]. Each party (also referred to as a country) that has ratified the WHO FCTC is provided with a reporting instrument. This instrument is used to report their progress on the implementation of the WHO FCTC articles [34]. A single reporting instrument was adopted in November 2010 to facilitate country reports. The reporting instrument is segmented into core mandatory questions and additional voluntary questions on the FCTC implementation guidelines. More details on the reporting instrument can be found on the WHO FCTC website.

### 2.4. Data Charting Process and Items

The authors extracted information from twenty peer-reviewed papers using a developed template informed by the Joanna Briggs Institute (JBI) data-extraction questions for scoping reviews [35]. The data-extraction questions covered key areas such as: author(s), year of publication, aim of study, settings, study population, study design, area/type of health communication intervention, and involvement of young people in the intervention design. The extracted data items were recorded on a table in a Microsoft Word document and saved on the personal computer of the first author. 

A pre-determined extraction template was used to extract relevant data from the WHO GHO ATMCs and the WHO FCTC Article 12 Reports.

**Figure 2:**
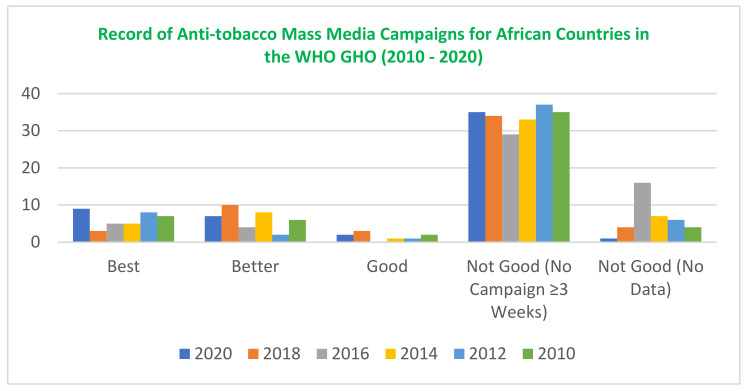
Record highlighting number of African countries conducting high-quality campaigns.

A. WHO Global Health Observatory database—The data-extraction items covered include: African country, World Bank Income group of African Country, Number of Campaigns Recorded (2010–2020), WHO ATMCs Category, Review Score, and Overall Review Score. Summaries of the extracted data is further highlighted in Figure 2 and Table 2, while the full list for 54 countries is in Appendix A, Table A2.
B. WHO FCTC Article 12 Reports—Number of Parties (Countries) that implemented Article 12, Year of WHO FCTC Report, Average % Implementation Rate of Article 12, % of Parties Focused on Health Risk of Tobacco Consumption, % of Parties Targeting Children, Stakeholders Involved in Implementation of Programs, and African Country Mentioned in Year of Report. A summary of the extracted data is highlighted in Appendix B.

### 2.5. Data Synthesis

Narrative data synthesis, informed by the Joanna Briggs Institute (JBI) manual for evidence synthesis [30,35], was conducted to comprehensively describe the methods and findings of the included studies. The types of health communication interventions and the target population of those interventions were compared across data sources to identify common themes in tobacco control health communication in Africa. Also, the studies were compared to explore whether young people were involved in the process.

## 3. Results

The authors identified 1725 studies from the database search. After duplicates were removed, 300 articles were screened. After the final review, 20 peer-reviewed studies were included. For the grey sources, data for 54 African countries were included along with information from six WHO FCTC Reports.

### 3.1. Study Characteristics

#### 3.1.1. Peer-Reviewed Sources

Most studies focused on a descriptive account of mass-media campaigns and the use of Health Warning Labels (HWLs). The studies that focused on mass-media campaigns using either traditional media or social media were twelve. These studies were conducted across eighteen African countries including: Senegal, Nigeria, Kenya, Ghana, Burkina Faso, Ethiopia, Liberia, Lesotho, Malawi, Swaziland, Uganda, Zambia, Zimbabwe, Egypt, Ethiopia, Mauritius, Tunisia, and Somaliland. Among the twelve studies, two made use of a mixed-methods design [36,37], five examined secondary data from national sources that applied a cross-sectional design [38,39,40,41,42], three made use of single and multiple cross-sectional surveys [21,43,44], while two made use of quasi-experimental designs [20,45].

The studies that focused on Health Warning Labels (HWLs) were six. These studies were conducted across four African countries including: Tunisia, Egypt, Ghana, and Nigeria. Among the studies, one made use of a mixed-methods design [46], four made use of single and multiple cross-sectional surveys [47,48,49,50], while one made use of a qualitative design [51].

Two studies focused on professional medical communications [52] and constituency relations [53]. The study on professional medical communications focused on reaching doctors using text messages to enable them to deliver cessation support to patients who were 12 years and above. The study that focused on constituency relations emphasized gaining the views of tobacco retailers as key stakeholders for effective tobacco control. Both studies were conducted in Nigeria and made use of pre-post design and cross-sectional design, respectively.

In total, seventeen studies had a preventive focus while three studies had a cessation goal [37,41,52]. The aforementioned three studies aimed at using health communication interventions like text messages and television advertisements, to propel medical personnel to engage in cessation treatment and to examine quit attempts. A summary of the peer-reviewed sources are shown in Table 3.

#### 3.1.2. Grey Sources

Between 2010 and 2020, the WHO GHO recorded anti-tobacco mass-media campaigns for fifty-four African countries [32].

In 2020, among the 54 countries recorded, only 9 conducted a campaign considered high-quality by the WHO based on the set criteria (see Table 1). In the same year, 35 countries could not conduct a national campaign that lasted for more than three weeks. The WHO GHO ATMCs records show that most African countries have consistently been unable to conduct campaigns that lasted for more than three weeks.

The authors awarded scores based on the category and quality of the recorded campaigns, with countries scoring seven and above highlighted (see Appendix A). These countries include: Cote d’Ivoire, Egypt, Ethiopia, Ghana, Mauritius, Morocco, Rwanda, Seychelles, Togo, and Tunisia. Tunisia had the highest score (14), with three campaigns of high quality (3 × 3= Best), two campaigns of medium quality (2 × 2 = Better), and one campaign of low quality (1 × 1 = Good). Although over the years countries like Tunisia, Togo, Seychelles, and Ghana have recorded at least three high-quality campaigns that applied ≥7 (plus TV/radio) of the characteristics, the majority of African countries have not been consistent in engaging high-quality mass-media campaigns as a health communication approach.

Also, between 2010 and 2021, the average implementation rate of the WHO FCTC Article 12 among countries went from 70% to 92%, but only nine African countries were mentioned cumulatively in the six reports. This further substantiates the records in the WHO GHO showing that Africa countries are not maximizing anti-tobacco campaigns and are not significantly impacting the high implementation rate of the WHO FCTC Article 12.

**Table 2:** Record of the average number of African countries conducting high-quality campaigns.

Number of African Countries						Total Number
Year of Campaign Recorded	Best (3) National Campaign with ≥7 CTS (Plus TV/Radio)	Better (2) National Campaign with ≤7 CTS (No TV/Radio)	Good (1) National Campaign with ≤4 CTS	Not Good (0) No National Campaign ≥ 3 Weeks	Not Good (0) No Data Reported	
2010	7	6	2	35	4	54
2012	8	2	1	37	6	54
2014	5	8	1	33	7	54
2016	5	4	0	29	16	54
2018	3	10	3	34	4	54
2020	9	7	2	35	1	54
Average	6	6	1	33	6	

Table highlighting the average number of countries that have conducted high-quality campaigns between 2010–2020.

**Table 3:** Characteristics of sources and citation.

Author/Year	Setting	Area/Type of Health Communication Addressed	Aim of Study	Study Design/Method	Population (Age/N Size)	Involvement of Young People in Intervention Design
Hutchinson et al. [20]	Ghana	Mass media Magazine, movies, a radio program, social media, and other promotional activities	Impact evaluation of SKY Girls, a youth-focused smoking-prevention and empowerment campaign targeting girls in Ghana.	Quasi-experimental matched design	2625 13–16-year-old girls	N/A
Karletsos et al. [21]	Ghana	Mass media, new media, and interpersonal communication Social media and mass media (blogs, magazines), group meetings and events (SKY Girls campaign)	Investigate how well anti-smoking messages, delivered through both mass media and social media, can help change how adolescents in urban Ghana think about the dangers of smoking, in a more positive direction.	Quantitative study	First wave of (7054) 3775 adolescent girls and 3279 adolescent boys aged 13–16 years in urban areas of Accra. Second wave of 5069 participants.	Not involved in design Minimally involved in implementation
Perl et al. [36]	Senegal, Nigeria, and Kenya	Mass-media campaigns Mass media 5 radio and 5 TV anti-smoking advertisements	Adapt available anti-tobacco television and radio advertisements from high-income countries for African countries.	Mixed-methods study	1078 male and female adult smokers and non-smokers 18–40 years	Not involved in design Other tobacco-control stakeholders involved in adaptation before study
Wakefield et al. [37]	From 10 LMICs—Bangladesh, China, Egypt, India, Indonesia, Mexico, Philippines, Russia, Turkey, and Vietnam	Mass media Five television advertisements	Examine the comprehension, acceptability, and how effective five television advertisements could be in conveying an anti-smoking message and encouraging adults in low- and middle-income countries to quit smoking.	Mixed-method study	2399 smokers aged 18–34 years	Not involved in design
Achia [38]	Burkina Faso, Ethiopia, Liberia, Lesotho, Malawi, Swaziland, Uganda, Zambia, and Zimbabwe	Mass-media campaigns Television, radio, newspapers, or magazines	Study the relationship between self-reported tobacco use and frequency of mass-media utilization in nine LMICs in Sub-Saharan Africa.	Quantitative cross sectional design using secondary data analysis from DHS	159,462 Women aged 15–49 years (n = 101,316) and men aged 15–59 years (n = 58,146)	N/A
Azagba et al. [39]	Mauritius	Mass-media campaign (sponge) Television advertisements	Examine the combined effect of increase in cigarette excise tax and anti-tobacco mass-media campaign (sponge) on smoking behavior.	Quantitative—longitudinal Study International Tobacco Control Mauritius Survey, 2009–2011 using secondary longitudinal data analysis	725 adult respondents, smokers and non-smokers (aged ≥18 years)	N/A
Bekalu et al. [40]	Ethiopia	Mass media Television, radio, billboards, posters, newspapers, magazines, movies	Examine if tobacco risk perceptions varied across socioeconomic and urban vs. rural population subgroups, and whether and how exposure to anti-smoking messages was associated with disparities in risk perceptions across socioeconomic and urban-rural subgroups.	Quantitative cross-sectional survey using secondary data analysis from GATS Ethiopia 2016	10,150 Male/female 15 years and above	N/A
Owusu et al. [41]	14 LMICs including Nigeria and Egypt (2009–2012)	Mass media Newspapers or magazines, television, radio, and billboards	Evaluated factors associated with three stages of intention to quit tobacco smoking among adults in 14 LMICs by using the transtheoretical model (TTM) of health behavior change (precontemplation, contemplation, and preparation).	Quantitative cross-sectional Secondary data analysis of publicly available GATS data from 14 LMICs from 2009 to 2012	43,540 current tobacco smokers aged 15 years and above	N/A
Siziya et al. [42]	Somaliland	Mass media Television, radio, billboards, posters, newspapers, magazines, and movies	To estimate the prevalence of cigarette smoking and determine associations of anti-smoking messages with smoking status.	Quantitative cross sectional survey using secondary data analysis from GYTS Somaliland 2004	1563 students 13–15 years	N/A
Oyapero et al. [43]	Lagos, Nigeria	N/A Anti-tobacco messages (ATM)	Assess the association between exposure to anti-tobacco messaging (ATM) and quit attempts among adolescents and young adults in Lagos, Nigeria.	Quantitative study	947 participants 15–35 years	N/A
Khalbous and Bouslama [44]	Tunis, Tunisia	Media Visual (paper) advertisements	To understand the relationship between smoking socialization and the effectiveness of anti-tobacco advertisements.	Quantitative—panel Surveys	351 students 12–16 years	Not involved in design
Odukoya et al. [45]	Lagos, Nigeria	Mass media Health talks, information leaflets, and posters	To assess the effect of a short school-based anti-smoking program on the knowledge, attitude, and practice of cigarette smoking among students in secondary schools in Lagos State.	Quantitative—non-randomized, controlled intervention	1031 students 10–21 years	Not involved in design Information leaflets and posters designed and introduced by researcher
Mansour et al. [46]	Tunisia	Media HWLs	Improve and adapt a set of 16 pictorial Water pipe specific health warning labels (HWLs) created in an international Delphi study, to the Tunisian context	Mixed Methods Study	63 young adults 18-43 years	Not Involved in Design
Adebiyi et al. [47]	Igbo-Ora, Nigeria	Media Graphic health warnings	To examine if the use of graphic health warnings can be effective in preventing smoking initiation among young people in Nigeria	Quantitative cross-sectional study	(554) students aged 13–17 years	Not involved in design
Borzekowski and Cohen [48]	Brazil, China, India, Nigeria, Pakistan, and Russia	Media Text/image Health-warning labels (HWLs)	Investigate the awareness and understanding of health-warning labels among 5- and 6-year-old children in six countries.	Quantitative survey	2423 5–6 year old	Not involved in design
Mostafa et al. [49]	Egypt	Media HWLs	Investigate whether PHWs on water-pipe tobacco products lead to behavior change.	Quantitative study	2014 water-pipe smokers and non-smokers aged 18 years or older	Not involved in design
Mostafa et al. [50]	Egypt	Media Water-pipe warning labels (WTP WL)	Measure the perceived efficacy of existing against novel enhanced (generic and water-pipe-specific) WTP WLs and the associated factors among Egyptian waterpipe smokers and non-smokers.	Quantitative design	2014 male and female waterpipe smokers and non-smokers ≥18 years	Not involved in design
Singh et al. [51]	Kumasi, Ghana	Media Text and pictorial health warnings	Examine how Ghanaian smokers and non-smokers view warning labels (text and pictures) on cigarette packs and to investigate their opinions regarding the implementation of pictorial warnings in Ghana.	Qualitative study	(85) 50 smokers and 35 non-smokers aged 15 years and older	Not involved in design
Odukoya et al. [52]	Nigeria	Professional medical communications Text messaging	Improve text messaging as an intervention among physicians to help them foster tobacco treatment (cessation) among their patients. Focal patients at least 12 years.	Quantitative study	(n946) Respondents = 165) In 3 tertiary care hospitals Age of medical personnel not mentioned	N/A
Uchendu et al. [53]	Nigeria	Constituency relations	To examine retailer awareness of tobacco-control laws and willingness to be involved in control activities.	Quantitative—cross-sectional	218 participants >30 ≥50 years	N/A

Table highlighting the peer-reviewed sources of evidence and extracted items.

### 3.2. Participant Characteristics

Twelve studies [20,21,36,37,38,39,40,41,42,43,44,45] that focused on mass-media campaigns recorded male and female participants who were smokers and non-smokers aged 10–59 years cumulatively. The participants in these studies were either conveniently, purposely, or randomly sampled or recruited from local groups, schools, or communities, based on the study’s purpose. 

Six studies [46,47,48,49,50,51] that focused on Health Warning Labels (HWLs) included male and female smokers and non-smokers aged 5–43 years cumulatively. The participants in these studies were purposely recruited or sampled from universities, hospitals, brothels, primary and secondary schools, and neighborhoods located in rural and urban areas.

Two studies [52,53] that focused on professional medical communications and constituency relations included participants who were doctors from tertiary-care hospitals and mostly female tobacco retailers aged >30 and ≥50 years, respectively. The participants in both studies were purposely recruited or sampled from a hospital and two communities that were rural and semi-rural.

Although most studies recruited participants aged 10–59 years, except in the studies by Karletsos et al., Khalbous and Bouslama, and Odukoya et al. [21,44,45], the health communication interventions were not mentioned to specifically target young people. Of the three studies, only Karletsos et al. [21] targeted and involved young participants in the implementation and even had some materials available online (see YouTube—SKY Girls GH). While Khalbous and Bouslama, and Odukoya et al. [44,45] targeted young people, none mentioned involving young participants in designing the materials used. The materials used by Odukoya et al. [45] were designed by the researcher, while Khalbous and Bouslama [44] did not mention the origin of the (paper) advertisements used in the study.

The health communication interventions from the 2018 WHO FCTC reports highlighted the use of the World No Tobacco Day (WNTD) as a key platform for tobacco control campaigns in countries [33] (p. 37). The campaigns were reported to have targeted young people and other population sub-groups. The reports did not emphasize if specific campaigns targeting young people 10–24 years were conducted, but in 2018 Chad reported implementing a peer-education training program for young people. In the same year, Nigeria recounted using social media (#ClearTheAir) and influential celebrity leaders in the entertainment industry to engage young people. Similarly, among the twenty studies highlighted, only one referred to involving young Ghanaian girls of 13–15 years in implementing the intervention [21]. The other nineteen studies did not mention engaging young people as stakeholders or key decision makers in designing or implementing interventions.

### 3.3. Study Findings

#### 3.3.1. Content and the Presentation Matters

Many studies found that content and the presentation of the content played a pivotal role in tobacco control health communication. Four studies noted that hard-hitting and emotionally evocative content presented using visual channels performed better among their participants. Perl et al. [36] found that the television advertisements, “Coughing Child and Baby Alive”, which focused on the consequences of smoking on vulnerable and innocent children, performed better than anti-tobacco industry messages among their participants aged 18–40 years. Mansour et al. [46] found that HWLs showing external health effects like oral cancer and the harmful effects on children were rated as more effective than those illustrating scientific facts about vague chemicals by their participants aged 18–43 years. Similarly, Wakefield et al. [37] found that the television advertisements featuring graphic images of serious health harms which elicited negative emotions and discomfort were perceived to be more effective. The advertisement was perceived as more effective than the one illustrating a medical term with the use of a complex metaphor, “Plastic Bubblewrap”, to explain smoking-related emphysema. Their participants were aged 18–40 years. Likewise, Adebiyi et al. [47] found that pictorial health warnings (PHWs), showing that smoking cigarettes can cause cancer of the airways and impotence, induced fear. The health warnings were thought to be better placed to prevent smoking initiation than those focused on stroke causation and harm to children. The participants in the study were 13–17 years. Although not related to emotionally evocative content, Siziya et al. [42] found that content and mode of delivery could affect effectiveness and could contribute to unintended consequences. Siziya et al. [42] suggested this was because exposure to anti-smoking messages through health workers and print media (posters, billboards, newspapers, and magazines) was not associated with reported smoking status, while exposure through sports or community events seem to be associated with reported smoking. The participants in the study were 13–15 years.

#### 3.3.2. Reaction to Anti-Tobacco Content Varies

The reaction to anti-tobacco content by different groups varies and this was emphasized by studies in this review. In most studies, the reaction of non-smokers to anti-tobacco content was more positive than that of non-smokers. Perl et al. [36] reported that non-smokers tended to rate advertisements in a more positive light than smokers. Mostafa et al. [49] similarly found that pictorial health warnings (PHWs) had more effect on non-smokers than smokers. The same study recounted that non-smokers understood and discussed PHWs considerably more with people than current and former water-pipe smokers. In their study on socialization, Khalbous and Bouslama [44] also found that anti-smoking advertising was not equally effective among children. The authors found that it was easier to convince those who already have a negative attitude towards smoking (anti-smokers) to never start smoking than those who have favorable attitudes (pro-smokers).

In a similar vein, Owusu et al. [41] reported that smokers who were 15–24 years old had higher chance of being in the preparation to quit stage than smokers aged 25 years and above. The age disparity was also noted in the study by Oyapero et al. [43], where younger participants were more likely to respond to anti-tobacco messages (ATMs) than older participants. Likewise, the study by Adebiyi et al. [47] reported that graphic warning labels depicting stroke as an effect of smoking were deemed less fearful by the young participants who most likely found it hard to engage with the subject as stroke was envisioned as a remote issue more associated with older persons.

#### 3.3.3. Anti-Tobacco Messages Can Influence Smoking

Many studies also found that anti-tobacco media messages could prevent smoking initiation, and promote intention to quit and quit attempts. Odukoya et al. [45] found that anti-smoking awareness programs were effective at improving tobacco-related health knowledge, influencing attitudes towards tobacco use and increasing the desire to quit among current adolescent smokers. Oyapero et al. [43] found significant association between exposure to anti-tobacco messages and quit attempts and that likelihood of quit attempts decreased with older age. Achia [38] also found a positive association between cigarette smoking and media utilization but that only a small percentage of women 15–49 years had access or utilized media facilities in Sub-Saharan Africa. Likewise, Bekalu et al. [40] found that exposure to anti-smoking messages was associated with greater risk perceptions of smoking, which could facilitate prevention of smoking initiation and intention to quit. Similarly, Azagba et al. [39] found that cigarette tax increases in combination with anti-tobacco national media campaigns impacted quit attempts, as the consumption of cigarettes significantly reduced. Siziya et al. [42] also found that knowledge related with having discussed with family or being taught in school about the harms of smoking was associated with reported non-smoking.

#### 3.3.4. Message Clarity and Contextual Considerations

Studies also found that messages need to be clear and context specific. Perl et al. [36] found that unambiguous messages that were culturally relevant had the ability to resonate with the target audience, which will in turn contribute to a successful campaign. Mansour et al. [46] also found that the type of message that was deemed important by tobacco control experts in a Delphi Study by Asfar et al. [54], was not deemed so by the study participants. They reported that HWLs that illustrated scientific facts without showing a specific disease were not deemed effective as they were unclear and hindered the acceptance of the message. Similarly, Singh et al. [51] found that participants considered text-only warnings to be ineffective in communication of the health risks of smoking and that picture and text warnings need to be context and country specific. Likewise, Odukoya et al. [52] found that sending a simple 160-character text message was effective in raising awareness among doctors to offer cessation treatment to patients.

## 4. Discussion

The available source of evidence shows a dearth in literature that comprehensively addresses all six areas of health communication as defined by Schiavo [11] (pp. 26–28). This is not surprising, given the reportedly low prevalence of tobacco use in Africa, which may not necessitate comprehensive research. Again, limited research designs, owing to a lack of research capacity that has been recorded in tobacco control within Sub-Saharan Africa over the last fifty years [55], could also be a pointer. No paper addressed policy communication, policy advocacy, community mobilization, and citizen engagement, while only one paper each addressed constituency relations and professional medical communication. The lack of studies that address policy communication, policy advocacy, community mobilization, and citizen engagement is an area that requires closer examination in Africa especially given the politics and power-play that often surrounds tobacco control [22,55]. 

It is also pertinent to mention that the focus of tobacco control health communication in the reported studies, was mostly preventive rather than cessative. Again, this is expected, given the low prevalence of tobacco use in the African region. Although the use of health communication to drive quit attempts has been established, this was not explored by most studies in the review. This could be due to a lack of a skilled clinical workforce, and inadequate resources to effectively manage cessation programs. The necessity for investment in tobacco control research to build capacity has been underscored as a critical need to support the evidence base needed for implementation of the WHO FCTC [55]. The limited evidence signals the need for more studies that cover a variety of areas to holistically address tobacco control health communication in Africa. Another probable reason for the dearth of literature on tobacco control health communication, especially among young people, could be the perspective ascribed to smoking. Smoking among adults is culturally and societally more acceptable than smoking among young people [56,57,58], who are not expected to practice the habit or identify as smokers. This age disparity creates a vacuum that will limit tobacco control intervention and research among young people. This is because involving young people in tobacco control could indirectly be acknowledging that young people also smoke, whether acknowledged by the adults or not.

The main area of health communication focused on was mass-media communications, and even that was not addressed effectively based on the set standard by the WHO FCTC (See Table 1). The limited use of high-quality mass media for health communication in tobacco control has also been highlighted in the 2023 WHO report on the global tobacco epidemic [59]. The report identified inadequate use of health communication among the countries of the world that may have been further exacerbated by the COVID-19 pandemic. The report stated that “less than one-quarter of the world’s 1.5 billion population were exposed to a best-practice mass-media campaign in the past two years, and people in low-income countries were the least exposed to anti-tobacco mass media” [59]. The limited use of high-quality health communication could be due to the varied barriers and limitations that currently impact tobacco control health communication [25,26]. One of the barriers (funding) could be why most countries align their health communication plan to the WHO World No Tobacco Day, and as such, have no country-specific long-term health communication plan or intervention. The issue arising from funding could also be why tobacco control has not enjoyed media coverage compared to tobacco-industry corporate social responsibility projects [60], as the tobacco industry wields more financial capacity to influence the narrative via the available media apparatus [23].

Again, although most studies focused on mass-media communication, when looked at closely, they were mostly secondary studies. These studies were not able to provide detailed information on the type of content or medium of presentation for anti-tobacco campaigns and messages when targeting young people. There is need to qualitatively ascertain the content that better suits young people based on their perspective, especially as mass-media campaigns and advertising bans have been identified as the most effective policies in Africa when applied singularly and mixed [61].

The content and medium of presentation of anti-tobacco messages holds a vital place in tobacco control health communication. For instance, the Global Progress Report between 2020 and 2021 [33], curated details on the number of WHO FCTC parties that focused on the health risk of tobacco consumption. Also, keen attention was paid to segregating campaigns that either engaged or did not engage the use of TV/radio in the WHO GHO ATMCs. Similarly, the studies that focused on mass-media campaigns and the use of Health Warning Labels (HWLs) also aimed to find the best possible content and means of presenting it in the best way for the participants, to achieve the desired outcomes. Participants in the studies were given the opportunity to identify their preferred anti-tobacco communication materials [36,47], so as to better fit the context and population. The focus on content and medium of presentation is not unusual, given the body of evidence that supports targeted messages for effective communication [14,15,62] and using a wide range of communication channels to reach a target group for an extended period [21,62]. The use of targeted messages and a wide range of communication channels to facilitate effective communication is even more critical when young people are the focal group, as they are still in the developmental phase of identifying their preferences and could be reached through a variety of channels and content [21].

Whereas content and presentation of anti-tobacco messages is relevant in health communication, the studies that considered mass-media campaigns and the use of health warning labels (HWLs) focused more on adapting and testing content than development. One of the advantages cited for focusing on adaptation is the cut down on scarce resources that would have been spent on development. These scarce resources are argued to be better managed when redirected to implementation and increasing the reach of and exposure to anti-tobacco content [36]. While this is a salient point, given the limited resources available for health communication in LMICs in Africa [6], development of content is still vital [39]. It is vital to reach underserved, overlooked, or hard to reach groups [52,63], enhance message novelty effect [40], and acknowledge the differences in reaction among different groups [36,49]. The message novelty effect enables the message recipient to pay more attention as people usually favor novel stimuli over familiar stimuli [64]. In addition, the disparity in reaction to content among different groups substantiates the need for target groups to be involved in the design and development of anti-tobacco content [36,49], as well the need to use health communication early [43,44,47] and consistently [62]. Health communication that takes into consideration the disparity in reaction to content is also important, as non-smokers and young people will react more positively than smokers and older people [36,49]. For this reason, the use of pictorial or graphic HWLs only on tobacco products at point of sale should be reconsidered, as a different audience could be reached and react better in a different setting, which will further maximize the benefits of health communication.

Whether adapting content for a specific context or developing new content, the relevance and clarity of the content are important. The ability of the target audience to process, comprehend, and accept the anti-tobacco message will impact the effectiveness of tobacco-control health communication. This is because messages that are clear, resonate with the audience, and are culturally and contextually relevant will contribute to a successful anti-smoking campaign [36,49]. The SKY Girls campaign is an example of an intervention that was evaluated as successful [20]. The intervention, though specifically adapted for young Ghanaian girls 13–15 years, made no mention of involving young people in the design of the intervention. This was because the intervention was being replicated in an African country after recording success in a high-income country. While the intervention did not engage the young people in its design, it encouraged them to participate in the implementation and to give their opinions. For instance, young girls were encouraged to come to events and bring their friends and were also engaged to provide their opinions about the SKY Girls film and activities. Another factor that contributed to the success of the SKY Girls campaign is the use of interpersonal relationships. The value of interpersonal relationships cannot be over-emphasized in tobacco control health communication when the focal group are young people, as they play a vital role. For instance, the study by Karletsos et al. [21] showed that message dissemination and reach improved when anti-tobacco messages were discussed among peers rather than when discussed with parents. The conversations with peers also impacted the risk perception of cigarettes and shisha via mass media and social media.

The involvement of the target audience has been highlighted as important in the process of development in health communication because what the target group sees as important may not be seen in the same way by experts [46]. Although most studies engaged the participants by using quantitative or qualitative approaches to gain their opinion, it was more consultative than participatory. Like Perl et al. [36], other studies [20,46] did not highlight any initial involvement of the focal groups in the design of the materials tested. However, they all pointed to the significance of engaging target groups in tobacco control health communication, as it was pivotal in ensuring communication is clear and relevant to the focal group. Although a consultative approach may be suitable when HWLs are the focus, given the rigorous need to match labels to professional, regulatory, and country-specific standards, it may not be the most suitable when developing mass-media campaign messages, especially as young people may require a more qualitative approach and outlook [14]. This lack of involvement of the focal groups at the onset may have been due to funding, accessibility, and time restraints, given that Perl et al. [36] only engaged professional and governmental tobacco control stakeholders in the process of adapting the communication materials.

Although the health communication interventions from the WHO FCTC reports highlighted that various stakeholders were involved or engaged in the interventions, young people were conspicuously absent from the list of stakeholders (See Appendix B). From the list of stakeholders reported, stakeholders like public agencies, NGOs, and international organizations were involved in the development and implementation of intersectoral programs and strategies for tobacco control. The stakeholders highlighted were those with some element of power or control, who could influence the desired campaign outcomes. While this is a good strategy given the vital role of NGOs and government bodies in tobacco control [61,65], it may not be the best approach with young people. As the perspective of young people has a strong chance of being different [49], it is important that they participate [46] in the initial stages of the process. This will ultimately save time and resources as the communication intervention would be relevant and appropriate. The drive to ensure a wide range of stakeholders are involved in tobacco control is encapsulated in the updated Global Alliance for Tobacco Control (GATC) Strategic Plan for 2022 to 2025 (Convening Stakeholders and Partners). The GATC plan [66] seeks to significantly incorporate more stakeholders within and outside the tobacco control space for effective and sustained implementation of the WHO FCTC.

### 4.1. Future Directions

The findings of this review signal a vacuum in the input of young people in Africa’s tobacco control health communication agenda. While young people are involved in the development of health communication materials, they are absent in the design process, which makes the activity more consultative than participatory. In the Global Progress Report, for instance, even though the percentage of parties that focused on the health risks of tobacco smoking increased from 70% to 92% between 2010 and 2021, it was not clear whether the health risk focused on aligned with the health priorities identified by the target group themselves. Also, in the Global Progress Report, while most countries consistently reported targeting children and young people, it was also not clear how they involved them in the design and development of the programs, or the materials used. Between 2010 and 2021, only two African countries in the WHO FCTC report (Chad, Nigeria) mentioned some level of participation by young people.

There is now a need for young people, who make up about 60% of the continent’s inhabitants [67], to be more involved in tobacco control health communication, as those not exposed to anti-smoking education are susceptible to using tobacco products [56,68]. Also, young people should be more involved in tobacco control if mass media and social media are the preferred channels for dissemination [21], as these platforms will play significant roles in the changing landscape of tobacco control by widening reach and influencing the narrative [69]. The involvement of young people will give room for targeted health communication that is more relevant and better incorporates local perspectives, which has been recommended as a best practice for tobacco control in Africa [70].

The available data also reflects the need for a more consistent, strategic, and targeted approach to health communication by African countries, especially given the rapid changes associated with tobacco, nicotine, and related products [71]. Although health communication alone will not solve the issue of tobacco smoking, it is still a vital part in every effective tobacco control program [12], and local perspectives must be engaged for success [49,70].

The prevalence of tobacco use in the African region is still small compared to other WHO regions; as such, having a strategic health communication plan that is driven by the perspective of Africans for Africans will be vital to ensure tobacco-use prevalence remains low. The programs and policies adopted must reflect the context for the associated interventions to be useful and sustainable. African parties of the WHO FCTC must take cognizance of their context, including identifying targeted content (focusing on groups), local structures, and resources, while integrating recommendations for effective health communication interventions. By taking cognizance of their contextual needs, African countries would lay the foundation for consistent and effective health communication interventions for Africans by Africans.

### 4.2. Limitations

The broad classification of health communication offered by Schiavo enabled the inclusion of other vital peer-reviewed papers, which would have been left out if a narrow classification had been used. While the WHO GHO provided data on mass-media campaigns, Schiavo’s classification enabled the authors to cover all areas of health communication.

The authors did not consider the use of HWLs as one of the forms of health communication interventions while examining the grey sources. This is because primary research has extensively covered health warnings in peer-reviewed sources. To limit selection bias, the authors did not exclude peer-reviewed papers that focused on testing developed health warnings with a population, as it would also give insight into participant involvement. Also, HWLs are attached to the tobacco product itself and are disseminated at the point of sale. In addition, Article 12 of the WHO FCTC and the WHO ATMC criteria do not touch on HWLs as this is covered in Article 11.

Article 12 of the WHO FCTC has 12 indicators. These indicators do not necessarily align with the MPOWER indicator ’W’, which covers ATMCs as well as HWLs simultaneously. To reduce this limitation, the author extracted data from the WHO GHO specific to anti-tobacco campaigns in addition to information on Article 12 from the Global Progress Reports. In addition, the Global Progress Report was first released in 2006, but this review started with the reports from 2010 as they align with the timeline (2010–2020) of the data reported in the WHO GHO ATMCs.

This review does not account for individual reports from each country for the Global Progress Reports. It only accounts for information provided by the WHO FCTC, who serve as a collating body for countries who are the primary source of information. The WHO FCTC, as the collating body, requests additional information on implementation by parties where necessary, to support the data provided and further enhance its relevance.

Lastly, this review, being a scoping review, does not uncover international evidence as it is specific to the African context; however, it exposes knowledge gaps in areas of health communication that need to be addressed in future research. It also incorporated data from an international body (WHO) that categorized and used evidence relevant globally. The fact that the WHO categorizes campaigns with a distinct set of criteria provides strong reasons for its inclusion in this review. The authors acknowledged these standards by incorporating papers and data that were related to it, with a special focus on the involvement of focal populations. 

## 5. Conclusions

This review has contributed to examining and highlighting the different areas of health communication in Africa, which could be vital for future studies. It has highlighted the dearth of tobacco control health communication literature and emphasized the lack of participation in tobacco control health communication among young people. Tobacco control health communication in Africa is still practiced inconsistently despite having the lowest prevalence in comparison to other WHO regions. This inconsistency can be addressed by leveraging local perspectives, local structures, and available resources and actively involving young people, who make up most of the African population, while integrating recommendations for effective health communication interventions. 

## Figures and Tables

**Table 1:** WHO GHO anti-tobacco mass-media campaign categories.

WHO GHO Category
5 = National campaign with ≥ 7 CTS (plus TV/radio)
4 = National campaign with ≤ 7 CTS (no TV/radio)
3 = National campaign with ≤ 4 CTS
2 = No national campaign ≥ 3 weeks
1 = No data reported
**CTS = Characteristics** The characteristics (CTS) of a high-quality campaign as enumerated by the WHO include: Campaign is part of a tobacco control program. Formative research to understand the target audience. Campaign materials are pre-tested with the target audience and refined in line with campaign objectives. Airtime (radio, television) and/or placement (billboards, print advertising, etc.) is obtained. Collaboration with journalists to gain publicity or news coverage. Process evaluation to assess how effectively the campaign had been implemented. Outcome evaluation to assess the campaign impact.

Table showing the WHO GHO categories and CTS of a high-quality campaign.

## Data Availability

Data are available in a publicly accessible repository that does not issue DOIs. Publicly available datasets were analyzed in this study. This data can be found here: https://www.who.int/data/gho/data/indicators/indicator-details/GHO/gho-tobacco-control-anti-tobacco-mass-media-campaigns (accessed 16 June 2023).

## Appendix A

**Table A1:** PRISMA-ScR (Preferred Reporting Items for Systematic Reviews and Meta-Analyses Extension for Scoping Reviews) checklist.

SECTION	ITEM	PRISMA-ScR CHECKLIST ITEM	REPORTED ON PAGE
**TITLE**			
Title	1	Identify the report as a scoping review.	1
**ABSTRACT**			
Structured summary	2	Provide a structured summary that includes (as applicable): background, objectives, eligibility criteria, sources of evidence, charting methods, results, and conclusions that relate to the review questions and objectives.	1
**INTRODUCTION**			
Rationale	3	Describe the rationale for the review in the context of what is already known. Explain why the review questions/objectives lend themselves to a scoping review approach.	3
Objectives	4	Provide an explicit statement of the questions and objectives being addressed with reference to their key elements (e.g., population or participants, concepts, and context) or other relevant key elements used to conceptualize the review questions and/or objectives.	2
**METHODS**			
Protocol and registration	5	Indicate whether a review protocol exists; state if and where it can be accessed (e.g., a Web address); and if available, provide registration information, including the registration number.	3
Eligibility criteria	6	Specify characteristics of the sources of evidence used as eligibility criteria (e.g., years considered, language, and publication status), and provide a rationale.	4
Information sources	7	Describe all information sources in the search (e.g., databases with dates of coverage and contact with authors to identify additional sources), as well as the date the most recent search was executed.	3, 5
Search	8	Present the full electronic search strategy for at least 1 database, including any limits used, such that it could be repeated.	3
Selection of sources of evidence	9	State the process for selecting sources of evidence (i.e., screening and eligibility) included in the scoping review.	3, 4
Data charting process	10	Describe the methods of charting data from the included sources of evidence (e.g., calibrated forms or forms that have been tested by the team before their use, and whether data charting was done independently or in duplicate) and any processes for obtaining and confirming data from investigators.	6
Data items	11	List and define all variables for which data were sought and any assumptions and simplifications made.	6
Critical appraisal of individual sources of evidence	12	If done, provide a rationale for conducting a critical appraisal of included sources of evidence; describe the methods used and how this information was used in any data synthesis (if appropriate).	N/A
Synthesis of results	13	Describe the methods of handling and summarizing the data that were charted.	6
**RESULTS**			
Selection of sources of evidence	14	Give numbers of sources of evidence screened, assessed for eligibility, and included in the review, with reasons for exclusions at each stage, ideally using a flow diagram.	4
Characteristics of sources of evidence	15	For each source of evidence, present characteristics for which data were charted and provide the citations.	9–12
Critical appraisal within sources of evidence	16	If done, present data on critical appraisal of included sources of evidence (see item 12).	N/A
Results of individual sources of evidence	17	For each included source of evidence, present the relevant data that were charted that relate to the review questions and objectives.	13
Synthesis of results	18	Summarize and/or present the charting results as they relate to the review questions and objectives.	13–15
**DISCUSSION**			
Summary of evidence	19	Summarize the main results (including an overview of concepts, themes, and types of evidence available), link to the review questions and objectives, and consider the relevance to key groups.	15–18
Limitations	20	Discuss the limitations of the scoping review process.	18
Conclusions	21	Provide a general interpretation of the results with respect to the review questions and objectives, as well as potential implications and/or next steps.	19
**FUNDING**			
Funding	22	Describe sources of funding for the included sources of evidence, as well as sources of funding for the scoping review. Describe the role of the funders of the scoping review.	19

**Table A2:** WHO Global Health Observatory anti-tobacco mass-media campaigns for 54 African countries, 2010–2020.

Country	World Bank Income Group	Number of Campaigns Recorded (2010–2020)	Best (3) National Campaign with ≥7 CTS (Plus TV/Radio)	Better (2) National Campaign with ≤7 CTS(No TV/Radio)	Good (1) National Campaign with ≤4 CTS	Not Good (0) No National Campaign ≥ 3 Weeks	Not Good (0) No Data Reported	Overall Review Score
Algeria	Low and middle income	6	0	0	0	2014 (0) 2012 (0) 2010 (0)	2020 (0) 2018 (0) 2016 (0)	0
2. Angola	Low and middle income	6	2020 (3)	0	0	2016 (0) 2014 (0) 2012 (0) 2010 (0)	2018 (0)	3
3. Benin	Low and middle income	6	0	2014 (2) 2012 (2)	0	2020 (0) 2018 (0) 2010 (0)	2016 (0)	4
4. Botswana	Low and middle income	6	0	2020 (2) 2010 (2)	2018 (1)	2016 (0) 2014 (0)	2012 (0)	5
5. Burkina Faso	Low and middle income	6	0	0	0	2020 (0) 2018 (0) 2016 (0) 2014 (0) 2012 (0) 2010 (0)	0	0
6. Burundi	Low and middle income	6	0	0	0	2020 (0) 2018 (0) 2014 (0) 2012 (0) 2010 (0)	2016 (0)	0
7. Cameroun	Low and middle income	6	2016 (3) 2014 (3)	0	0	2020 (0) 2012 (0) 2010 (0)	2018 (0)	6
8. Cape Verde (also called Cabo Verde)	Low and middle income	6	2020 (3)	2018 (2)	0	2016 (0) 2014 (0) 2012 (0) 2010 (0)	0	5
9. Central African Republic	Low and middle income	6	0	2014 (2)	0	2020 (0) 2018 (0) 2010 (0)	2016 (0) 2012 (0)	2
10. Chad	Low and middle income	6	0	2018 (2)	0	2020 (0) 2014 (0) 2012 (0) 2010 (0)	2016 (0)	2
11. Comoros	Low and middle income	6	0	0	0	2020 (0) 2018 (0) 2016 (0) 2014 (0) 2010 (0)	2012 (0)	0
12. Congo	Low and middle income	6	0	0	0	2020 (0) 2018 (0) 2016 (0) 2014 (0) 2012 (0) 2010 (0)	0	0
13. Cote d’ Ivoire	Low and middle income	6	0	2020 (2) 2018 (2) 2016 (2) 2014 (2)	2010 (1)	2012 (0)	0	9
14. Democratic Republic of Congo	Low and middle income	6	0	0	0	2020 (0) 2018 (0) 2016 (0) 2012 (0)	2010 (0) 2014 (0)	0
15. Djibouti	Low and middle income	6	0	2014 (2)	0	2020 (0) 2018 (0) 2012 (0) 2010 (0)	2016 (0)	2
16. Egypt	Low and middle income	6	2012 (3) 2010 (3)	0	2020 (1)	2018 (0) 2016 (0) 2014 (0)	0	7
17. Equatorial Guinea	Low and middle income	6	0	0	0	2020 (0) 2014 (0) 2012 (0) 2010 (0)	2018 (0) 2016 (0)	0
18. Eritrea	Low and middle income	6	0	2010 (2)	0	2020 (0) 2018 (0) 2014 (0) 2012 (0)	2016 (0)	2
19. Eswatini (also called Swaziland)	Low and middle income	6	0	0	0	2020 (0) 2018 (0) 2012 (0) 2010 (0)	2016 (0) 2014 (0)	0
20. Ethiopia	Low and middle income	6	2020 (3)	2018 (2) 2016 (2)	2012 (1)	2014 (0) 2010 (0)	0	8
21. Gabon	Low and middle income	6	0	0	0	2020 (0) 2018 (0) 2016 (0) 2014 (0) 2012 (0)	2010 (0)	0
22. Gambia	Low and middle income	6	0	2020 (2) 2018 (2) 2014 (2)	0	2016 (0) 2012 (0) 2010 (0)	0	6
23. Ghana	Low and middle income	6	2020 (3) 2014 (3) 2012 (3)	2018 (2)	0	2016 (0) 2010 (0)	0	11
24. Guinea	Low and middle income	6	0	2010 (2)	0	2020 (0) 2018 (0) 2016 (0) 2014 (0) 2012 (0)	0	2
25. Guinea-Bissau	Low and middle income	6	0	0	0	2020 (0) 2018 (0) 2016 (0) 2012 (0) 2010 (0)	2014 (0)	0
26. Kenya	Low and middle income	6	2016 (3)	0	0	2020 (0) 2018 (0) 2014 (0) 2012 (0) 2010 (0)	0	3
27. Lesotho	Low and middle income	6	0	2018 (2)	0	2020 (0) 2014 (0) 2012 (0) 2010 (0)	2016 (0)	2
28. Liberia	Low and middle income	6	2012 (3)	2020 (2)	0	2018 (0) 2014 (0) 2010 (0)	2016 (0)	5
29. Libya	Low and middle income	6	2014 (3)	0	0	2020 (0) 2018 (0) 2016 (0) 2012 (0) 2010 (0)	0	3
30. Madagascar	Low and middle income	6	2012 (3) 2010 (3)	0	0	2020 (0) 2018 (0) 2016 (0) 2014 (0)	0	6
31. Malawi	Low and middle income	6	0	0	0	2020 (0) 2018 (0) 2014 (0) 2012 (0) 2010 (0)	2016 (0)	0
32. Mali	Low and middle income	6	0	0	0	2020 (0) 2018 (0) 2016 (0) 2014 (0) 2012 (0) 2010 (0)	0	0
33. Mauritania	Low and middle income	6	0	0	0	2020 (0) 2018 (0) 2014 (0) 2012 (0)	2016 (0) 2010 (0)	0
34. Mauritius	Low and middle income	6	2016 (3) 2012 (3)	2014 (2)	0	2020 (0) 2018 (0) 2010 (0)	0	8
35. Morocco	Low and middle income	6	2020 (3) 2016 (3) 2010 (3)	2018 (2)	0	2014 (0) 2012 (0)	0	11
36. Mozambique	Low and middle income	6	0	0	0	2020 (0) 2018 (0) 2016 (0) 2014 (0) 2012 (0) 2010 (0)	0	0
37. Namibia	Low and middle income	6	2020 (3)	2018 (2)	0	2016 (0) 2014 (0) 2012 (0) 2010 (0)	0	5
38. Niger	Low and middle income	6	2010 (3)	0	0	2020 (0) 2018 (0) 2016 (0) 2014 (0)	2012 (0)	3
39. Nigeria	Low and middle income	6	0	0	0	2020 (0) 2018 (0) 2012 (0) 2010 (0)	2016 (0) 2014 (0)	0
40. Rwanda	Low and middle income	6	2020 (3) 2010 (3)	2012 (2)	2018 (1)	2016 (0) 2014 (0)	0	9
41. Sao Tome & Principe	Low and middle income	6	2012 (3)	0	0	2020 (0) 2018 (0) 2010 (0)	2016 (0) 2014 (0)	3
42. Senegal	Low and middle income	6	2018 (3) 2014 (3)	0	0	2020 (0) 2016 (0) 2012 (0) 2010 (0)	0	6
43. Seychelles	High income	6	2018 (3) 2016 (3) 2012 (3)	2020 (2)	2010 (1)	2014 (0)	0	12
44. Sierra Leone	Low and middle income	6	0	2016 (2)	0	2020 (0) 2018 (0) 2014 (0) 2012 (0) 2010 (0)	0	2
45. Somalia	Low and middle income	6	0	0	0	2020 (0) 2018 (0) 2016 (0) 2012 (0)	2014 (0) 2010 (0)	0
46. South Africa	Low and middle income	6	0	2020 (2)	0	2018 (0) 2016 (0) 2014 (0) 2012 (0) 2010 (0)	0	2
47. South Sudan	Low and middle income	6	0	2010 (2)	0	2020 (0) 2018 (0) 2016 (0)	2014 (0) 2012 (0)	2
48. Sudan	Low and middle income	6	0	2014 (2) 2010 (2)	0	2020 (0) 2018 (0) 2016 (0) 2012 (0)	0	4
49. Togo	Low and middle income	6	2020 (3) 2018 (3) 2010 (3)	2014 (2)	0	2016 (0) 2012 (0)	0	11
50. Tunisia	Low and middle income	6	2020 (3) 2014 (3) 2012 (3)	2016 (2) 2010 (2)	2018 (1)	0	0	14
51. Uganda	Low and middle income	6	0	2020 (2) 2018 (2)	2014 (1)	2016 (0) 2012 (0) 2010 (0)	0	5
52. United Republic of Tanzania	Low and middle income	6	0	0	0	2020 (0) 2018 (0) 2014 (0) 2010 (0)	2016 (0) 2012 (0)	0
53. Zambia	Low and middle income	6	2010 (3)	0	2020 (1)	2018 (0) 2016 (0) 2014 (0) 2012 (0)	0	4
54. Zimbabwe	Low and middle income	6	0	0	0	2020 (0) 2018 (0) 2016 (0) 2014 (0) 2012 (0) 2010 (0)	0	0

## Appendix B

**Table A3:** Global Progress Report on the Implementation of the WHO FCTC 2010–2021 (Article 12: African Parties).

Number of Countries (Parties) That Reported Implementing Article 12	Year of WHO FCTC Report	Average % Implementation Rate of Article 12	% of Parties Focused on Health Risk of Tobacco Consumption	% of Parties Targeting Children	Stakeholders Involved in Implementation of Programs	African Country Mentioned in Year of Report
114	2010	Not mentioned	80	Not given (4 out of 5)	Public agencies and Non-governmental organizations not affiliated with the tobacco industry.	None
115	2012	70	100	98	Public agencies and non-governmental organizations, private organizations, religious and faith-based organizations, academic and higher education institutions, community and scientific groups, and professional colleges, as well as international organizations and bodies (page 34).	Ghana Training of Healthcare Professionals by the Ministry of Health (page 33) Djibouti Unavailable resources for impactful campaigns (page 35)
125	2014	70	100	99	Public agencies and NGOs, private organizations, religious and faith-based organizations, academic and higher education institutions and hospitals, community and scientific groups, professional colleges, municipalities, the media, and international organizations, including WHO. (Page 35).	Senegal Launch of first ever anti-tobacco media campaign called “Sponge” (page 34)
119	2016	90	100	99	Public agencies and non-governmental organizations involved in development and implementation of intersectoral programs and strategies for tobacco control. Private organizations, academic and higher educational institutions, community and scientific groups, professional colleges, municipalities, the media, and international organizations, including WHO (page 35/36).	Seychelles launch or culmination of national program planned to align with World No Tobacco Day (page 34)
162	2018	99	99	99	Public agencies and NGOs involved in the development and implementation of intersectoral programs and strategies for tobacco control. Academic and higher education institutions, community and scientific groups, hospitals and research institutes, professional colleges, police and military, the media, and international organizations, including WHO. (pages 40/41).	Chad New campaign on oral cancer (page 37) Training young peer educators in smoking prevention (page 40) Nigeria Launched campaign called “#ClearTheAir” to support new smoke-free legislation (page 39) Malta Training of local administrators and police officers after smoking ban in cars when minors are present (page 40) Either established comprehensive national tobacco-control communications strategy/action plan or in the process of developing one (page 41)
166	2021	92	99	96	Public agencies and NGOs, academic and higher education institutions, community and scientific groups, professional colleges, police and the military, the media, and international organizations, including WHO, were involved in the development and implementation of intersectoral programs and strategies for tobacco control (page 52).	Senegal Continued or further developed previously established campaigns/activities (page 47) World No Tobacco Day (WNTD) campaign (page 47) Mauritius Continued or further developed previously established campaigns/activities (page 47)

This table shows WHO FCTC parties between 2010 and 2021 with extracted items for Article 12.
